# OPA1 drives macrophage metabolism and functional commitment via p65 signaling

**DOI:** 10.1038/s41418-022-01076-y

**Published:** 2022-10-28

**Authors:** Ricardo Sánchez-Rodríguez, Caterina Tezze, Andrielly H. R. Agnellini, Roberta Angioni, Francisca C. Venegas, Chiara Cioccarelli, Fabio Munari, Nicole Bertoldi, Marcella Canton, Maria Andrea Desbats, Leonardo Salviati, Rosanna Gissi, Alessandra Castegna, Maria Eugenia Soriano, Marco Sandri, Luca Scorrano, Antonella Viola, Barbara Molon

**Affiliations:** 1 Department of Biomedical Sciences, University of Padova, 35131 Padova, Italy; 2 Istituto di Ricerca Pediatrica IRP- Fondazione Città della Speranza, 35127 Padova, Italy; 3 Veneto Institute of Molecular Medicine, 35129 Padova, Italy; 4 Clinical Genetics Unit, Department of Women’s and Children’s Health, University of Padova, Padova, Italy; 5 Department of Biosciences, Biotechnologies and Environment, 70125 Bari, Italy; 6 Department of Biology, University of Padova, 35131 Padova, Italy; 7 Department of Medicine, McGill University, Montreal, Montreal (Quebec) H4A 3J1 Canada

**Keywords:** Cell death and immune response, Inflammation

## Abstract

Macrophages are essential players for the host response against pathogens, regulation of inflammation and tissue regeneration. The wide range of macrophage functions rely on their heterogeneity and plasticity that enable a dynamic adaptation of their responses according to the surrounding environmental cues. Recent studies suggest that metabolism provides synergistic support for macrophage activation and elicitation of desirable immune responses; however, the metabolic pathways orchestrating macrophage activation are still under scrutiny. Optic atrophy 1 (OPA1) is a mitochondria-shaping protein controlling mitochondrial fusion, cristae biogenesis and respiration; clear evidence shows that the lack or dysfunctional activity of this protein triggers the accumulation of metabolic intermediates of the TCA cycle. In this study, we show that OPA1 has a crucial role in macrophage activation. Selective Opa1 deletion in myeloid cells impairs M1-macrophage commitment. Mechanistically, Opa1 deletion leads to TCA cycle metabolite accumulation and defective NF-κB signaling activation. In an in vivo model of muscle regeneration upon injury, Opa1 knockout macrophages persist within the damaged tissue, leading to excess collagen deposition and impairment in muscle regeneration. Collectively, our data indicate that OPA1 is a key metabolic driver of macrophage functions.

## Introduction

Mitochondria are highly mobile and dynamic organelles that constantly undergo fission and fusion events. These processes, collectively named as mitochondria dynamics, impact on mitochondrial reactive oxygen species (ROS) levels, calcium homeostasis, and oxidative phosphorylation [1]. Fission favors the segregation of damaged mitochondria for subsequent degradation by selective autophagy (mitophagy) and facilitates mitochondrial transport in polarized cells. Fusion, conversely, ensures mitochondrial mixing and complementation, increases mitochondrial ATP production, and ultimately prevents mitochondria from being engulfed by nascent autophagosomes.

The mitochondrial fission/fusion machinery is controlled by “mitochondria-shaping” proteins including both pro-fission members, such as the cytosolic GTPase dynamin-related protein 1 (Drp1) and its outer mitochondrial membrane adapters hFis1, Mff, and Mid49-51; and pro-fusion members, such as the large dynamin-like GTPases Opa1 in the inner mitochondrial membrane (IMM) and mitofusin (Mfn) 1 and 2 in the outer mitochondrial membrane (OMM) [2]. Besides the crucial role in controlling mitochondrial fusion, OPA1 is involved in the stabilization of respiratory chain super complexes [3], the remodeling and maintenance of mitochondria *cristae* [4] finally contributing to the control of cell death, proliferation and the modulation of gene expression in fibroblast, cardiomyocytes, and neutrophils [3–5]. In addition, the occurrence of mutations in the gene encoding *OPA1* are associated with autosomal dominant optic atrophy (ADOA) and ADOA+ syndromes, characterized by visual loss and in the latter case also by more widespread neurological symptoms [6–8].

In the immune system, mitochondrial shape is intimately linked to multiple facets of both lymphoid and myeloid cell functions. In neutrophils and in T cells, mitochondria redistribution and shape rearrangement are required to regulate the cell motor of migrating lymphocytes. Mitochondrial fission promotes mitochondrial relocation to the uropod, facilitating cell migration, whereas mitochondrial fusion inhibits both relocation and migration [9]. Among the different mitochondria-shaping proteins, OPA1 appears to play a key role in both the lymphoid and myeloid subsets. Indeed, genetic ablation of *Opa1* in neutrophils limits their antibacterial activity by reducing the formation of extracellular traps in a mechanism that involves the reduction in glycolytic ATP production [10]. In the T-cell subset, *Opa1* deletion affects cristae dynamics, impairing the generation of long-term T-cell memory responses [11]. Deletion of *Opa1* early during T cell development substantiates its essential role for thymocyte maturation at the double negative (DN)3 stage. The consequences of Opa1 deletion are far reaching, as surviving Opa1-deficient T cells are skewed towards a constitutively effector memory phenotype but are metabolically unfit in the long term [12]. Altogether, these studies place an IMM protein surprisingly at the crossroad of multiple aspects of T cell and neutrophil biology.

Macrophages are the first line of response to homeostasis perturbation. They rapidly sense multiple cues from the surrounding environment, integrate signals and execute coordinated responses. Depending on the local milieu, macrophages can polarize toward a specific functional phenotype conventionally recognized as pro-inflammatory (M1) or anti-inflammatory (M2) [13]. Importantly, the switch between M1 and M2 states depends on their metabolic reprogramming [14]. At a major difference from the case of T cells, the impact of mitochondrial fission/fusion on macrophage metabolic reprogramming, polarization, and effector functions remains poorly defined. Upon pro-inflammatory activation, Mfn2 appears to be required for mitochondrial respiratory changes and to produce reactive oxygen species (ROS) [15]. Nonetheless, whether and how OPA1 participates not only in neutrophil and T-cell biology, but also in macrophage responses is unknown.

With this question in mind, we studied the role of OPA1 in macrophages.

## Results

### OPA1 deletion drives mitochondrial dysfunction and affects macrophage polarization

**Fig. 1:**
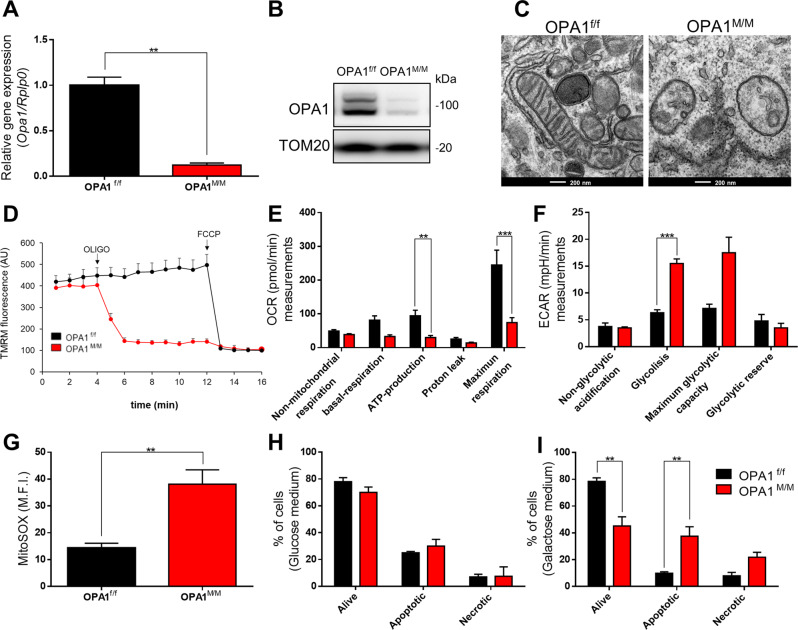
OPA1 deletion induces mitochondria dysfunction in macrophages. BMDMs were differentiated for 7 days with 40 ng/mL of MCSF. Relative OPA1 expression was evaluated by **A** RT-qPCR, using *Rplp0* as housekeeping and **B** western blot, representative image normalized with TOM20 in BMDM from OPA1^f/f^ and OPA1^M/M^ (*n* = *3*). **C** Representative pictures of Transmission Electron Microscopy analysis in OPA1^f/f^ and OPA1^M/M^ BMDM for mitochondria morphology and cristae. Scale bar 200 nm. (*n* = *3*) **D** Mitochondrial membrane potential (ΔΨ) was measured with TMRM (50 nM) by fluorescence microscopy. Images were acquired before and after FCCP (4 μM) addition. The graph reported the difference of fluorescence intensities before and after FCCP (*n* = *3*). Seahorse EFA analysis for **E** O_2_ consumption rate (OCR) and **F** Extracellular acidification rates (ECAR) were performed as indicators of OXPHOS and aerobic glycolysis respectively (*n* = *3*). **G** Reactive Oxygen Species (ROS) produced by mitochondria were measured with a MitoSOX probe by FACS analysis (*n* = *6*). Live/Death BMDMs were analyzed after culture in **H** glucose or **I** galactose medium with Annexin V assay. Graph represents % of cells (*n* = *3*). Data are represented as mean ± SEM. Statistical analysis was performed by Unpaired nonparametric *t* test (**P < 0.01, ***P < 0.001).

To investigate the role of OPA1 in macrophage functions, we generated a myeloid-conditional Knockout mouse, the *Opa1*^f/f^*Lyz2*^Cre/Cre^ (hereafter referred as *Opa1*^M/M^) by crossing *Opa1*^f/f^ mice [3] with mice expressing Cre recombinase under the control of the lysozyme 2 endogenous promoter (*Lyz2*^Cre/Cre^ mice). *Opa1*^f/f^ mice were used as controls in all the experiments. We confirmed Opa1 deletion in bone marrow-derived macrophages (BMDMs) by Real-Time quantitative PCR and Western Blot analysis (Fig. 1A, B). As expected, electron microscopy (EM) confirmed that mitochondria were fragmented, mitochondria cristae were enlarged and scarcer in *Opa1*^M/M^ BMDMs (Fig. 1C and Fig. SP 1A). These morphological changes were accompanied by mitochondrial dysfunction. Real-time imaging of mitochondrial membrane potential (Δψ_m_), using the potentiometric dye Tetramethylrhodamine (TMRM), revealed mitochondrial depolarization following ATP synthase inhibition by oligomycin in OPA1^M/M^ cells, compared to control ones (Fig. 1D), suggesting that in Opa1-deficient mitochondria of BMDMs the ATP synthase runs in reversal mode to sustain membrane potential [16] and to compensate for latent mitochondrial dysfunction. Indeed, in OPA1^M/M^ cells oxygen consumption rate (OCR) was lower (Fig. 1E). Despite an increase in glycolysis (Fig. 1F), the intracellular levels of ATP (Fig. SP 1C) and the NAD^+^/NADH ratio (Fig. SP 1D) were lower as compared to the control, and higher cytoplasmatic calcium (Fig. SP 1E) was found. We also detected a significant increase of mtROS in OPA1^M/M^ macrophages (Fig. 1G and Fig. SP 1B), as observed in other cell types [17], that could explain the latent mitochondrial dysfunction. Finally, these cells were addicted to glycolysis, as confirmed by the fact that their growth was reduced when cultured in galactose-supplemented/glucose-free medium (Fig. 1H, I and SP 2A). Taken together, our results indicate that OPA1 deletion impairs mitochondrial bioenergetics in resting macrophages. Of note, no significant differences in the apoptosis rate were observed between control OPA1^f/f^ and OPA1^M/M^ cells upon cell death induction by Staurosporin (Fig. SP 2B). Since the assembly of ETC respiratory supercomplexes (RCS) plays a key role in regulating innate immune functions, and, in particular, in macrophage responses to pathogens [18], we asked whether OPA1 deletion modulates RCS assembly. We first examined the effect of OPA1 deletion on RCS in resting (M0), LPS + IFNγ (M1) or IL-4 (M2) primed macrophages. Macrophage viability during cytokine-induced polarization was not affected by OPA1 deletion (Fig. SP3A, B). Given the glycolytic nature of OPA1^M/M^ cells, we further investigated the susceptibility to apoptosis of these cells, at different glucose concentrations. We did not detect differences in cell viability and apoptosis rate in OPA1^f/f^ and OPA1^M/M^ M0 and M2 cells upon cell death induction at varying glucose levels. However, we observed that OPA1^M/M^ M1 macrophages were ~10% more susceptible to cell death than M1 control cells, at both 11 and 5.5 mM glucose (Fig SP. 2C–E).

**Fig. 2:**
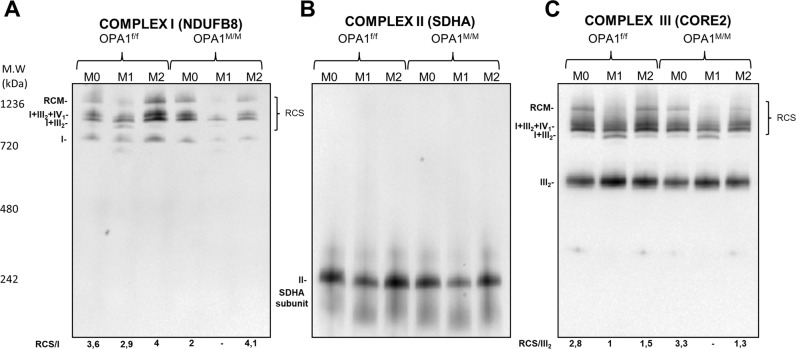
OPA1 deletion impairs mitochondria supercomplex assembly. OPA1^f/f^ and OPA1^M/M^ BMDMs were differentiated with MCSF (40 ng/mL) for 7 days, then BMDMs were treated with: M0: MCSF (10 ng/mL); M1: LPS (500 ng/mL) and IFNγ (25 ng/mL); M2: IL-4 (20 ng/mL) for 24 h. Electron transport chain supercomplex assembly (RCS) were examined by Blue Native Gel Electrophoresis in polarized macrophages (M0/M1/M2) for **A** Complex I (NDUFB8), **B** Complex II (SDHA) and **C** Complex III (Core2). Representative pictures (*n* = *3*).

In line with an impaired mitochondrial function (Fig. 1), the assembly of respiratory chain supercomplexes was defective in OPA1^M/M^ macrophages (Fig. 2A–C and Fig SP 3C).

### OPA1 deletion impairs macrophage polarization

**Fig. 3:**
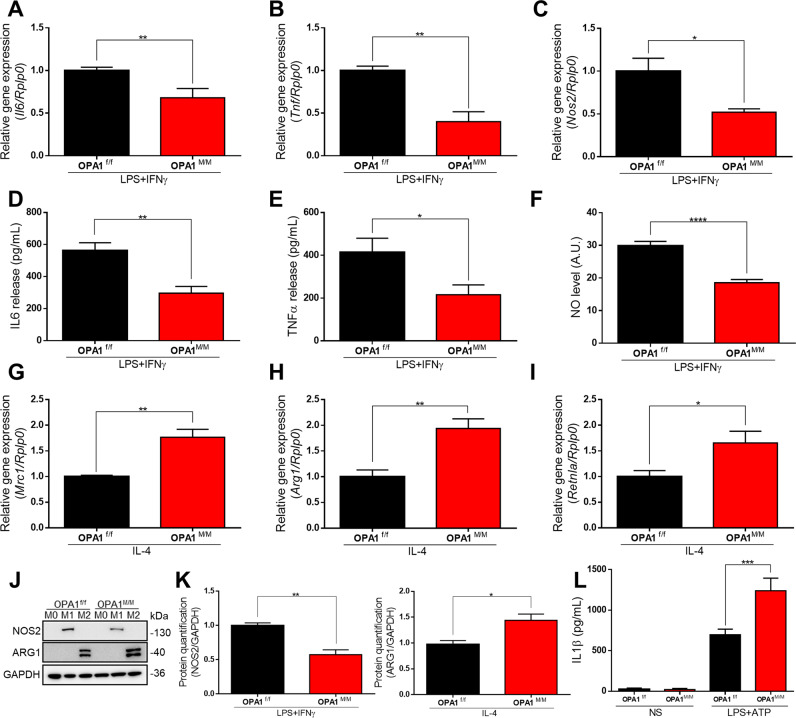
OPA1 deletion impairs macrophage polarization. OPA1^f/f^ and OPA1^M/M^ BMDMs were differentiated with MCSF (40 ng/mL) for 7 days, then BMDMs were stimulated with: M0: MCSF (10 ng/mL); M1: LPS (500 ng/mL) and IFNγ (25 ng/mL); M2: IL-4 (20 ng/mL) for 24 h. Relative gene expression for M1 polarization markers (**A**) Il6, (**B**) Tnf and **C** Nos2. Rplp0 was used as housekeeping in all the experiments (*n* = *5*). **D** IL6 and **E** TNFα protein levels were measured by ELISA in cell culture supernatants upon M1 polarization (*n* = *4*). **F** Nitric Oxide (NO) level quantification in M1 macrophages measured by DAR-4M AM staining. Relative gene expression for M2 markers (**G**) Mrc1 (**H**) Arg1 and **I** Retnla by real-time RT-PCR. Rplp0 was used as housekeeping in all the experiments (*n* = *5*). **J** Representative western blot and **K** quantification for NOS2 and ARG1 of total proteins (*n* = *5*). GAPDH was used as a loading control. Inflammasome activation was performed in M0 macrophages that were primed by 100 ng/ml LPS for 4 h and activated by ATP (5 mM, for the last 15 min) (**L**) ELISA of IL1β in the supernatants (*n* = *4*), no stimulated (NS) cells were used as a control. Data are presented as mean ± SEM. Statistical analysis was performed by Unpaired nonparametric *t* test (*P < 0.05, **P < 0.01, ***P < 0.001, ****P < 0.0001).

To evaluate the impact of OPA1 deficiency on macrophage polarization, we analyzed the expression levels of M1/M2 markers in BMDMs upon in vitro priming towards M1 (by LPS + IFNγ) or M2 (by IL-4). Remarkably, qPCR and WB analysis revealed that the expression of the pro-inflammatory genes Il6, Tnf, Nos2 and Ifnβ was significantly reduced in OPA1^M/M^ M1 macrophages (Fig. 3A–C, K and Fig. SP 4A), as compared to control M1 cells. Consistently, we also observed a decreased IL6 and TNFα release and a reduced NO burst in OPA1^M/M^ macrophages polarized towards M1, as compared to control cells. (Fig. 3D–F), indicating an inadequate performance of M1 macrophages caused by OPA1 deficiency. On the other side, the expression of conventional anti-inflammatory markers, such as Mrc1, Arg1 and Retnla was upregulated in M2 OPA1^M/M^ cells (Fig. 3G–I, K). As multiple studies have linked mitochondria defects and ROS generation to NLRP3 inflammasome activation [19, 20], we measured IL1β release in OPA1^M/M^ cells stimulated by LPS/ATP. Not surprisingly, we observed a significant increase in the release of IL1β in macrophages lacking OPA1, with respect to their control cells (Fig. SP 4B). Altogether, these data indicate that OPA1 deficiency causes major alterations in macrophage activation and polarization.

**Fig. 4:**
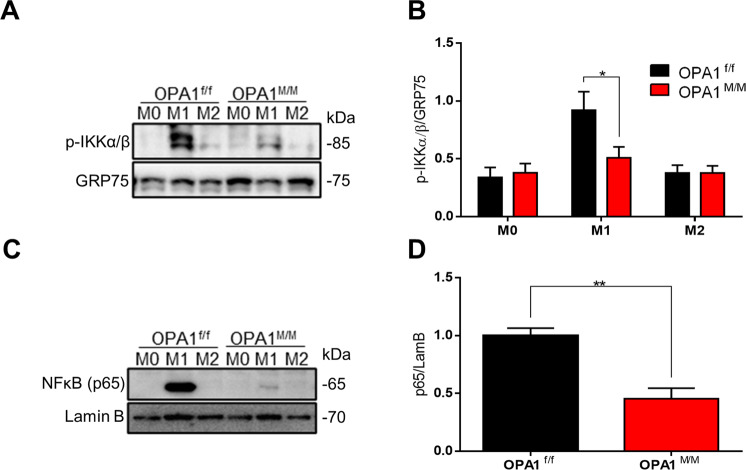
OPA1 deletion impairs NF-κB activation in macrophages. OPA1^f/f^ and OPA1^M/M^ BMDMs were differentiated with MCSF (40 ng/mL) for 7 days, then BMDMs were treated with: M0: MCSF (10 ng/mL); M1: LPS (500 ng/mL) and IFNγ (25 ng/mL); M2: IL-4 (20 ng/mL) for 24 h. **A** Representative western blot and **B** quantification in total proteins for phospho-IKKα/β and **C** Representative western blot and **D** quantification in nuclear extracts for NF-kB (p65) (*n* = *5*). GRP75 or Lamin B were used as a loading control. Data are presented as mean ± SEM. Statistical analysis was performed by Unpaired nonparametric *t* test (*P < 0.05, **P < 0.01).

We then sought to investigate the molecular ground underpinning the functional unbalance between the M1 and M2 macrophage phenotype due to OPA1 deficiency. NF-κB represents an essential driver of pro-inflammatory function in macrophages in response to LPS stimulation [18]. Because of the observed impairment of M1 commitment of OPA1^M/M^ macrophage, we asked whether defects in the NF-κB signaling were responsible for the impaired M1 commitment of OPA1^M/M^ macrophages. To do so, we investigated the NF-κB pathway in these cells. First, we analyzed the phosphorylation level of IKK, a kinase required for the activation of the canonical NF-κB pathway [21]. We detected a lower level of IKK phosphorylation in OPA1^M/M^ M1 macrophages triggered by LPS, as compared to control cells (Fig. 4A, B). Notably, OPA1 deletion did not impair LPS sensing in macrophages, as shown by the normal expression of the Toll-like receptor 4 (TLR4) in OPA1^M/M^ cells both in resting condition and after LPS stimulation (Fig. SP5). In addition to defective IKK phosphorylation, OPA1^M/M^ M1 macrophages displayed defective nuclear translocation of the RelA/p65 subunit in response to LPS (Fig. 4C, D and SP 6A, B), indicating that OPA1 deficiency influences NF-κB triggering, but not that of the typical M2 transcriptional factors STAT6 and PPARγ which were not altered by OPA1 deletion (Fig. SP 6C, D).

### OPA1 deletion affects macrophage M1/M2 skewing by altering the TCA cycle

Multiple lines of evidence indicated that M1 and M2 macrophages display different bioenergetic demands. Conventionally, the M1 phenotype rely on aerobic glycolysis for ATP generation and increased glucose and glutamine consumption. On the other side, M2 cells favor oxidative metabolism, especially FAO as the primary ATP source [13].

**Fig. 5:**
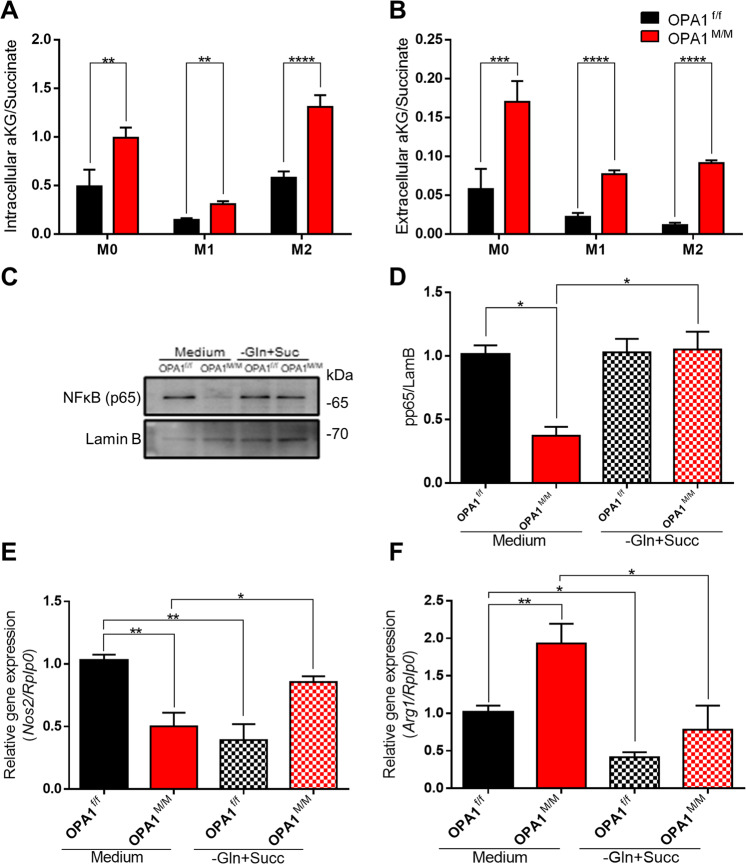
OPA1^M/M^ macrophages display metabolic features in accordance to NF-κB impairment. OPA1^f/f^ and OPA1^M/M^ BMDMs were differentiated with MCSF (40 ng/mL) for 7 days, then BMDMs were treated with: M0: MCSF (10 ng/mL); M1: LPS (500 ng/mL) and IFNγ (25 ng/mL); M2: IL-4 (20 ng/mL) for 24 h. Intracellular (**A**) and extracellular (**B**) 2-OG/succinate ratio quantified by LC-MS/MS analysis in M0, M1, and M2 macrophages. BMDM were differentiated to M1 or M2 in medium with Glutamine (Gln) 300 mg/L, Gln deprivation (-) or supplemented with Dimethylsuccinate (Succ/+) 5 mM. **C** Representative western blot and **D** quantification for p65 in nuclear fraction proteins (*n* = 4). Lamin B was used as a loading control. Relative gene expression analysis for **E** Nos2 and **F** Arg1 were performed by real-time RT-qPCR after M1 or M2 polarization in normal medium or medium supplemented with Succinate and Glutamine deprivation (*n* = *5*). Rplp0 was used as housekeeping. Data are presented as mean ± SEM. Statistical analysis was performed by Unpaired nonparametric t test (*P < 0.05, **P < 0.01, ***P < 0.001, ****P < 0.0001).

This paradigm could be reasonably applied for cells with a performing metabolic machinery. As we detected a metabolic unbalance in OPA1^M/M^ cells mainly due to an altered RCS assembly, we performed metabolic profiling of either resting M0 and M1/M2 primed OPA1^M/M^ macrophages. Consistently, we detected a defective TCA cycle in OPA1^M/M^ macrophages as compared to control cells with a consequent accumulation of multiple TCA intermediates (Fig. SP 7C–J), among which the extracellular succinate and α-ketoglutarate (αKG). TCA cycle intermediates have been identified as driving metabolites leading to macrophage commitment [22–24]. More recently, it has been also described that metabolic activities, in particular the glutamine handling, tune macrophage activation by acting on canonical signaling cascades and epigenetic reprogramming. Specifically, the αKG/succinate ratio differs in M1/M2 polarized macrophages being higher in M2 cells [25]. In line with this, we found that OPA1^M/M^ cells showed an increased αKG/succinate ratio as compared to control macrophages (Fig. 5A, B). With respect to the M1 phenotype, αKG is known to suppress the NF-κB pathway in LPS-stimulated mouse macrophages [25].

Indeed, we analyzed the expression level of either M1 (Nos2) or M2 (Arg1) markers by selectively modulating the αKG/succinate ratio during the M1/M2 polarization by culturing cells in medium with either Glutamine (Gln) or not (Gln−), and supplemented or not with Dimethylsuccinate (Succ/+) 5 mM. Importantly, when the polarization of OPA1^M/M^ macrophages to M1/M2 occurred under glutamine deprivation and succinate enrichment, the NF-κB activation was rescued (Fig. 5C, D) and NOS2 and ARG1 levels were similar to those expressed by control macrophages (Fig. 5E, F and SP 7A, B).

When the TLR4 signaling cascade leading to NF-κB activation was better analyzed in OPA1^M/M^ macrophages, we unveiled significant defects in TAK1, IkB, and p38 phosphorylation that could be rescued by glutamine deprivation and succinate administration (Fig. SP 8A–C).

Collectively, our data indicate that OPA1 deletion affects macrophage M1/M2 skewing by suppressing NF-κB signaling through a mechanism involving altered mitochondrial bioenergetics and TCA cycle flux.

### OPA deletion impairs muscle regeneration

**Fig. 6:**
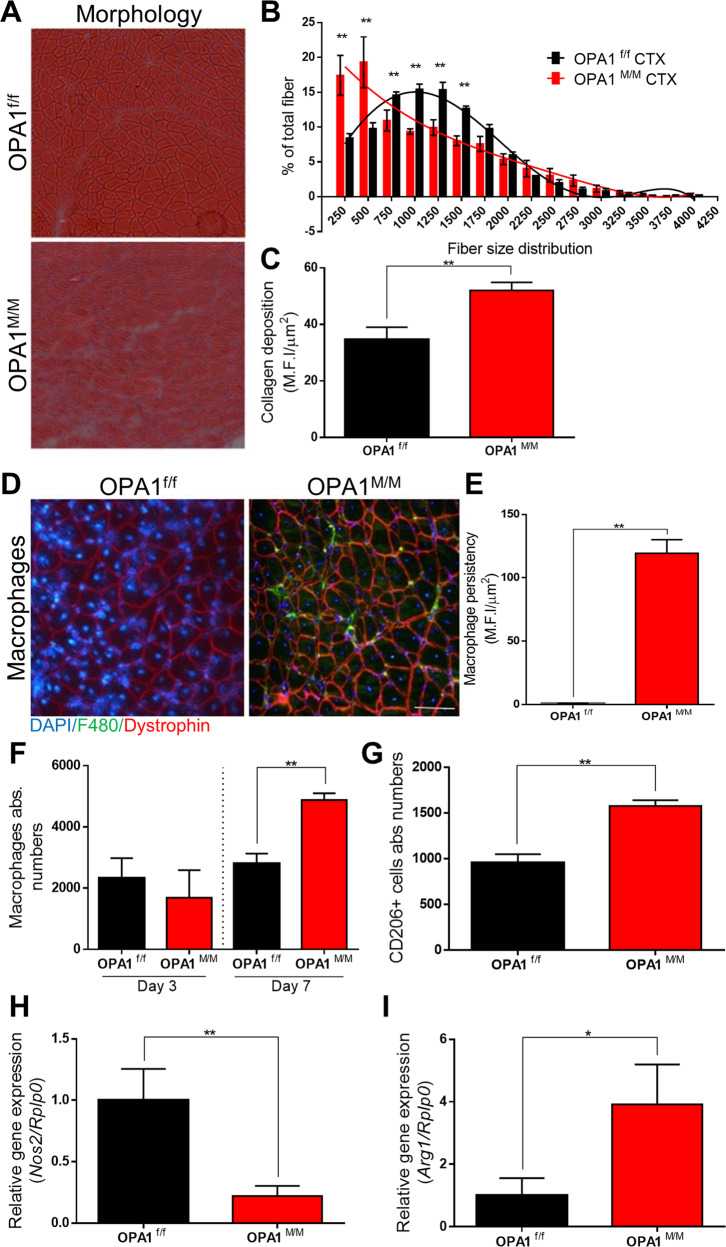
OPA1^M/M^ macrophages activation affects muscle regeneration. Muscle regeneration model was induced by CTX (35 μM) injection in the gastrocnemius, tibialis, and quadriceps muscles in OPA1^f/f^ and OPA1^M/M^ mice, tissue was collected at the day 3, 7 and 14 for the analysis. Tissue morphology at 14 DPI was analyzed by hematoxylin and eosin. **A** Representative pictures with objective 10X. Scale bar 100 μm. **B** Fiber size distribution (*n* = *3*) and **C** mean fluorescence of intensity of collagen staining (*n* = *3*). Quantification was performed with smash software in immunofluorescence for muscle fibers. **D** Representative pictures of macrophages immunofluorescence in the tissue at the 14 DPI. F4/80 (green), dystrophin (red) and nuclei were stained with Hoechst (blue). **E** Quantification of fluorescence intensity (*n* = *3*). **F** Fluorescence-activated cell sorting (FACS) analysis was performed on day 3 and 7 for macrophage quantification recruitment (*n* = *4*). **G** FACS analysis of CD206 positive cells at day 7 in muscle regeneration model (*n* = 4). Relative gene expression analysis for **H** Nos2 and **I** Arg1 were evaluated by real-time RT-qPCR (*n* = *4*). Rplp0 was used as housekeeping. Data are presented as mean ± SEM. Statistical analysis was performed by unpaired nonparametric *t* test (*P < 0.05, **P < 0.01).

We then addressed the physiological relevance of OPA1 deficiency in macrophage skewing and activity in vivo. To this aim, we took advantage of a murine model of muscle regeneration and healing after injury, a process in which the sequence and duration of the M1 and M2 macrophage gathering are crucial for proper tissue repair [26]. It is widely accepted that macrophages provide a specific microenvironment for satellite cell activation, proliferation, and differentiation, by sequentially adopting a pro- and anti-inflammatory profile. The functional switch from M1 toward the M2 phenotype occurring during muscle regeneration represents a central event for muscle healing. In particular, it has been recently shown that increasing the number of M1 macrophages after traumatic muscle injury boosts muscle recovery and reduces the fibrosis [27]. In our model, we induced myonecrosis by cardiotoxin (CTX) injection in the gastrocnemius, tibias and quadriceps muscles of either control or OPA1^M/M^ mice (Fig. SP 9A). Muscle tissue morphology and macrophage recruitment at the damaged sites were analyzed at day 3, 7 and 14 post-injury. Normally, ten days after injection, the overall tissue architecture is restored and most of myofibers display centrally located nuclei [28]. Interestingly, after 14 days of regeneration, we still detected morphological alterations in the muscles of OPA1^M/M^ mice (Fig. 6A–E) in terms of fiber distribution (Fig. 6A, B), higher collagen deposition (Fig. 6C), and macrophage persistency (Fig. 6D–E) and this underling a delay in regeneration process after injury. Therefore, to better characterize the functional properties of OPA1^M/M^ muscle infiltrating macrophages, we analyzed earlier time points upon muscle injury. FACS analysis of muscle cell suspension showed that at day 7 after CTX injection, a higher number of macrophages (Fig. 6F), but not granulocytes or monocytes (Fig. SP 9B), was present in the damaged muscle of OPA1^M/M^ mice compared to the control. Interestingly, the phenotypical profiling of these cells indicated that they expressed a higher level of the M2 marker CD206 (Fig. 6G and SP 9C), compared to macrophages from control mice. Real-time quantitative PCR analysis of muscle infiltrating macrophages, sorted at day 7 post-injury, showed that OPA1^M/M^ macrophages have a significant decrease in Nos2 expression (Fig. 6H) and concomitant increase in the level of Arg1, and pro-fibrosis and collagen-associated genes including Tgfβ, Col1a1, and Col3a1 (Fig. 6I and Fig SP 9D–F).

## Discussion

Over the last few years, multiple studies emphasized the critical role of mitochondrial dynamics in regulating innate and adaptive immune responses, focusing on their contribution to support cell energy demand [29]. Our group pioneered the field by unraveling a previously unexpected role for mitochondria morphological adaptations in the immune system, where they master movement of lymphocytes to chemoattractants [9]. More recently, the contribution of mitochondria dynamics to relevant immune processes including cell differentiation [30], activation [31] and metabolism [11] has been further elucidated. Currently, few reports have addressed the functional significance of mitochondria remodeling for innate myeloid cells.

In macrophages, mitochondrial fission is a critical process for the uptake of apoptotic bodies in vitro and efficient efferocytosis in vivo [32]. A recent study indicated that MFN2, but not MFN1, is required for ROS generation in macrophages and for the control of the inflammatory responses. Importantly, lack of MFN2 in vivo correlated with increased susceptibility to *Listeria monocytogenes* and *Mycobacterium tuberculosis* [15].

In neutrophils, the ablation of the pro-fusion protein OPA1 caused the remodeling of the mitochondrial cristae structure and impaired glycolysis efficiency by shortening NAD^+^ availability [10].

Our study was designed to clarify the role of OPA1 in macrophage commitment and functions. As previously observed in multiple cellular models, such as mouse embryonic fibroblasts (MEFs), skeletal muscle cells, hepatocytes, and cardiac cells [4, 33, 34], the absence of OPA1 resulted in a fragmented mitochondrial morphology in macrophages. Moreover, we found that the lack of OPA1 in primary macrophages triggers defects in mitochondrial membrane potential, reflecting a defective pumping out of proton from the matrix to the inner membrane space, as result of an unfit electron flow through the electron transport chain. These results are in line with previous data obtained in different cell types and showing that OPA1 stabilizes respiratory chain super complexes, sustains ATP production, maintains mitochondrial membrane potential, and prevents ROS generation [4, 17, 33, 35].

Bearing a remarkable impairment in mitochondrial bioenergetics, OPA1^M/M^ macrophages were addicted to glycolysis, as confirmed by their reduced growth when cultured in galactose-supplemented/glucose-free medium. Of note, OPA1^M/M^ cells displayed low levels of intracellular ATP and reduced NAD^+^/NADH ratio as compared to control cells. The shortening in the ATP/NAD^+^ amount confirms previous data obtained in OPA1 deficient neutrophils [10].

In line with previous reports using different cells [3, 36], our study pointed out a defective assembling of respiratory super complexes in OPA1^M/M^ macrophages. Defects in the Complex I and III have been linked to an increase in mtROS generation [37, 38] that we also observed in our cells. As well-recognized, mtROS fuel the NLRP3 inflammasome activation in macrophages [39]; in this line, we observed that the release of IL1β was significantly increased in OPA1^M/M^ cells with respect to the control ones. Of note, it has been previously reported that aberrant mitochondria elongation sustains NLRP3 inflammasome activation by facilitating NLRP3 mitochondrial localization and promoting NLRP3 expression [40]. In addition, multiple studies have linked the mitochondrial ETC to NLRP3 inflammasome activation through ROS generation [19, 20]. On the other hand, it has been also reported that the mitochondrial ETC sustains NLRP3 inflammasome activation via a ROS-independent mechanism [41]. So far, the role of mitochondrial dynamics in the regulation of inflammasome activation in macrophages is not completely understood. The observed enhanced inflammasome activation in OPA1-deficient macrophages may be the consequence of high ROS levels in these cells but may also involve other mechanisms and molecular interactions.

A peculiar feature of macrophages is their huge capability of sensing environmental stimuli and dynamically mounting the appropriate response by activating distinct molecular pathways. In addition to provide energy, mitochondrial metabolism has come out as an essential regulator of macrophage polarization and functions [42]. Conventionally, glycolysis and the pentose phosphate pathway (PPP) are preferentially exploited by the pro-inflammatory M1 macrophages, that present a broken Krebs cycle, to meet their ATP requirements. On the other hand, M2 macrophages mainly rely on fatty acid oxidation (FAO) and OXPHOS to support their metabolic demand [13]. This dogmatic view is progressively updating. Although still a matter of investigation, evidence indicated that glycolysis is also critical for the functional skewing to the M2 phenotype [43, 44]. Indeed, M2 macrophages appear more prone to flexibly adapt their bioenergetics programs depending on nutrient availability [45, 46]. Our data indicate that macrophages lacking OPA1 are biased towards undergoing glycolytic metabolism. On the other hand, OPA1^M/M^ cells showed a clear impairment toward the commitment to the pro-inflammatory M1 phenotype and, conversely, they presented typical traits of M2 cells both in vitro and in vivo. The deviation from the canonical metabolic reprogramming of M1/M2 macrophages that we observed in our setting might be determined by a functional compensation in OPA1^M/M^ cells, which show a defective performance of the metabolic machinery.

Solid evidence established the key role of OPA1 in the mitochondrial regulation of programmed cell death by apoptosis. The disruption of mitochondrial cristae structure caused by the absence of OPA1, facilitates the discharge of cytochrome c in response to apoptotic stimuli [4]. Consistently, *Opa1* deletion has been associated with an increased susceptibility to apoptosis in different cell types [12, 47]. In our model, we observed a reduced number of myeloid cells in vivo (data not shown) although we could not detect differences in cell viability and apoptosis rate in OPA1^f/f^ and OPA1^M/M^ in in vitro experiments. However, while performing ex vivo cytokine-driven macrophage polarization, we observed a specific susceptibility to cell death induction of M1, but not M0 or M2, macrophages, thus strengthening the idea that OPA1 deficiency mostly impacts on M1 cells.

The unbalanced M1/M2 commitment, caused by the selective ablation of OPA1 in macrophages, significantly delayed muscle regeneration and healing after injury. Indeed, 14 days after the induction of myonecrosis by CTX, we still detected morphological alterations in the muscles of OPA1^M/M^ mice in terms of fiber distribution, higher collagen deposition, and persistency of macrophages expressing higher levels of the M2 marker CD206 and Arg1 and pro-fibrosis and collagen-associated genes. The aforementioned susceptibility of OPA1^M/M^ M1 cells to apoptosis at physiological glucose level might contribute to the delay in the recovery of muscle integrity that we observed in vivo upon injury. While the impact of OPA1 ablation in muscle and satellite cells has been extensively investigated [33], to the best of our knowledge, this is the first report that investigates the contribution of OPA1 deficiency in the myeloid compartment during muscle regeneration.

The pro-inflammatory phenotype in macrophages is mainly regulated by the activation of the master transcription factor NF-kB [48]. However, several mechanisms including the activation of the NRF2-HO1 [49] axis and itaconate accumulation [50] have been related to defective NF-kB signaling. Interestingly, an additional mechanism by which macrophages integrate metabolic hints and epigenetic modification has been proposed [25]. Specifically, the authors revealed that αKG prevents M1 activation by suppressing IKKβ activation, via its PHD-dependent proline hydroxylation [23].

In our model, we uncovered upstream defects in the molecular way leading to NF-kB activation upon TLR4 stimulation, and in particular in the phosphorylation of TAK1, IkB, and p38. This defective signal could be clearly rescued by glutamine deprivation and succinate administration in macrophage cultures; it is indeed conceivable that the αKG/succinate ratio might affect this molecular way possibly by a post-translational regulation of protein interactors including the TAK1-TABs complex. Indeed, succinate and itaconate have both been shown to modify proteins ultimately tuning macrophage commitment and functions [51].

TCA cycle intermediates function as signaling molecules that regulate the mitochondrial-nuclear communication enabling a dynamic adaption of cell transcriptional profile in response to metabolic cues. Changes and accumulation of mitochondrial metabolites indeed drive nuclear gene transcription by controlling chromatin remodeling and epigenetic events that ultimately facilitate the positioning of defined transcription factors.

A recent report clearly defined a direct role of OPA1 in cell-autonomous adipocyte browning and identified the chromatin remodeling protein Kdm3a and urea cycle metabolites, including fumarate, as effectors of *Opa1*-dependent browning [52].

αKG is an essential cofactor of 2-oxoglutarate-dependent dioxygenases (2-OGDD), including the histone demethylases JMJDs and TET DNA demethylases, while succinate is the product of 2-OGDD enzymes reactions and thus, when it accumulates, it works as an antagonist of the reaction [53, 54]. In this line, we observed the accumulation of all Krebs cycle intermediates and a high ratio of αKG/succinate in OPA1^M/M^ macrophages. As previously mentioned, by altering the αKG/succinate ratio (i.e., by culturing cells under glutamine deprivation and succinate enrichment), we were able to rescue NF-κB activation and to bring back NOS2 and ARG1 levels to those expressed by control macrophages.

Additional mechanisms may play a role in the metabolic alterations observed macrophages lacking OPA1. The enzyme alpha-ketoglutarate dehydrogenase (OGDH) is allosterically activated by mitochondrial Ca^2+^ levels [55, 56] and OPA1 deletion is known to result in abnormalities in Ca^2+^ homeostasis and failure in mitochondrial Ca^2+^ handling [57, 58], suggesting a defective OGDH activity in OPA1^M/M^ cells. Of note, recent evidence indicates that changes in the mitochondrial and cytosolic Ca^2+^ content are linked to M1/M2 macrophage polarization [59].

Collectively, our findings provide further evidence for the role of OPA1 as master regulator of innate immunity. OPA1 deletion in macrophages impairs signaling, metabolic adaptations and functional polarization and results in a remarkable delay in muscle regeneration upon injury. Given the prominent role of macrophages in multiple pathological conditions, the OPA1-mediated metabolism pathway might represent a target for novel strategies to control inflammatory disorders. Pathogenic mutations in the *Opa1* gene are linked to the non-syndromic autosomal dominant optic atrophy (ADOA) [6, 60] and other syndromes bearing important neurological defects. Indeed, the development of novel molecules selectively and safely targeting OPA1 in specific cell compartments will be essential for the clinical exploitation of this protein.

## Materials and methods

### Animal procedures

Mice were maintained in the animal house of Venetian Institute of Molecular Medicine (VIMM). All experiments followed committee guidelines and the institutional protocols for animal care that was approved by Ministero della Salute (361/2018-PR). Mice without *Opa1*^f/f^*Lyz2*^Cre/Cre^ (hereafter referred as *Opa1*^M/M^) or *Opa1*^f/f^ genotype were excluded of the analysis. Mice were randomly allocated to treatment groups after age and sex matching. Mice used were 8-12 weeks old.

### BMDMs

Bone marrow-derived macrophages were obtained by flushing from murine (OPA1^f/f^ or OPA1^M/M^) femur and tibia and differentiated into macrophages in RPMI 1640, 10% FBS (Superior, Millipore), Glutamine (300 mg/L), sodium pyruvate (1 mM), 2-mercaptoethanol (0.01 mM), HEPES (25 Mm) in the presence of M-CSF (Miltenyi Biotec), 40 ng/ml for 5 days and then refilled with 2 mL more with M-CSF (20 ng/mL) for 2 more days. At day 7, cells were differentiated to: M1 with Lipopolysaccharide from E. coli O111:B4 (Sigma-Aldrich) (LPS) (500 ng/mL) and IFNγ, 25 ng/mL (Miltenyi Biotec) or M2 with IL-4 (Miltenyi Biotec) 25 ng/mL; for 24 h at 37 °C, 5% CO_2_. Different treatments were performed blinded to the mouse genotypes.

### RT-qPCR real time

A total of 0.5–1 × 10^6^ cells were used for RNA extraction. Total RNA was extracted with Trizol (Invitrogen) following the manufacturer’s protocol. The cDNA reactions were prepared from 500 ng of total RNA using the High Capacity cDNA Reverse Transcription Kit (Applied Biosystems) and a 1:10 dilution was used for quantitative PCR. The reactions were carried out using specific primers indicated in Supplementary Table 1 by SYBR Green in quantum real-time PCR system (Applied Biosystem). Data were normalized on the Rplp0 gene expression using the comparative ΔΔCt method [61].

### Western Blot

At least 2 × 10^6^ cells were used for total protein extraction with RIPA buffer while nuclear fractions were performed with 4 × 10^6^ cells following [62] protocol.

Protein extracts, 20 μg for total protein and 5 μg for nuclear extracts, were separated by 4–12% Bold NuPage (ThermoScientific) polyacrylamide gels and transferred onto PVDF 0.22 µm membranes (BioRad). Then membranes were processed with a blocking solution with Albumin (Sigma-Aldrich) 3% in TBS and incubated with primary antibodies (1:1000) listed in Supplementary Table 2, overnight at 4 °C. After that the membranes were incubated with an anti-rabbit peroxidase-conjugated secondary antibody (GE healthcare). Chemiluminescence was obtained by the ICL substrate (GE healthcare), and images were obtained with an imaging ImageQuant LAS 500 (GE healthcare).

### Electron microscopy

The samples were processed in the Electron Microscopy facility of the Dept. of Biology, University of Padova according to standard protocols. Briefly, 1 × 10^6^ BMDMs were fixed with 2.5% of glutaraldehyde and then maintained in a cacodylate buffer (0.1 M). Then the samples were treated with 1% of Osmium tetroxide and 1% of Potassium ferrocyanide, after that cells were dehydrated with ethanol and embedded in media for electron microscopy Epon (Sigma-Aldrich). Ultrafine (60-80 nm) dissections were performed with Ultratome V (Leica) ultramicrotome and contrasted with 1% of Uranyl acetate and 1% of Lead citrate. Images were obtained with transmission electron microscopy FEI Tecnai G^2^ (FEI) operating at 100 Kv and captured with Veleta (Olympus Soft Imaging System) digital camera.

### Seahorse

Metabolic parameters were calculated using the Seahorse XF24 as has been described in [59] and [63]. Briefly, BMDM from OPA1^f/f^ and OPA1^M/M^ were seeded and polarized in a 24-well Seahorse XF24 cell culture microplate in complete medium and maintained for 24 h at 37 °C, 5% CO_2_. To evaluate the Oxygen Consumption Rate (OCR) and extracellular acidification rate (ECAR), one hour previous to the experiment the medium was replaced with Seahorse medium (Dulbecco’s Modified Eagle Medium (DMEM) Sigma-Aldrich) supplemented with 33 mM NaCl, 1 mM sodium pyruvate, 15 mg/l phenol red, and 2 mM glutamine, pH 7.4 and sequentially injecting the following reagents: glucose (25 mM), Oligomycin A (1.5 µM), Carbonyl cyanide-4-(trifluoromethoxy)phenylhydrazone (FCCP) (1.6 µM), Antimycin A (2.5 µM) and Rotenone (1.25 µM). Data were analyzed with Aligent Seahorse Wave software.

### Mitochondrial membrane potential

Mitochondrial membrane potential assessment was measured by tetramethylrhodamine methylester (TMRM) accumulation. Cell fluorescence images were acquired with a Leica DMI6000B microscope upon incubation with serum-free media supplemented with 50 nM TMRM for 30 min. Data were analyzed using Metafluor software (Universal Imaging). 540 ± 20 nm excitation and 590 nm long-pass emission filter settings were used. Clusters of mitochondria were identified as regions of interest (ROI). To exclude artefacts due to different loading capacity of the cells, sequential images were acquired before and after carbonyl cyanide-p-trifluoromethoxy-phenylhydrazone (FCCP, 4 µM) addition, a protonophore that depolarizes mitochondria. ΔΨm was estimated as the difference in TMRM fluorescence intensity before and after FCCP of ROI from at least 30 cells.

### Mitochondrial ROS

ROS were detected by Mitosox (Thermo Fisher). A total of 1 × 10^6^ macrophages were seeded and stimulated with LPS 100 ng/mL, unstimulated cells were used as a control of basal ROS production. For the experiments, cells were incubated Mitosox (2.5 µM) for 30 min. Then cells were washed with Calcium/Magnesium free PBS, positive cell events for Mitosox were acquired with BD FACSCelesta. The mean fluorescence intensity of cells was quantified. Data were analyzed using FlowJo V10.0 Software.

### Mitochondria supercomplex analysis

Macrophage mitochondria were isolated by Digitonin permeabilization and differential centrifugation. Macrophage pellets were resuspended in Native Buffer (Thermo-Scientific) with 4% of Digitonin, after incubation were centrifuged at 16,000 g, the supernatants were collected and G250 sample buffer with 5% of G250 was added 1:3 to the samples. Then 50 µg of mitochondrial proteins were loaded and run on NativePAGE gel (Thermo-Scientific) and blotted.

### Mass spectrometry

To extract polar metabolites for mass spectrometry analysis, 5 × 10^5^ cells were scraped in 80% methanol and 100 µL of culture medium were suspended in 400 µL of 100% methanol. Subsequently, the samples were centrifuged and dried using a vacuum concentrator and stored at −80 °C. The measurements of the metabolites were obtained with an Acquity UPLC system interfaced with a Quattro Premier mass spectrometer (Waters, Milford, MA, USA) [64]. The calibration curves were established using standards and processed under the same conditions as the samples, at five concentrations [65, 66]. The best fit was determined using a regression analysis of the peak analyte area. The multiple reaction monitoring transitions selected in the negative ion mode were m/z 116.88 > 73.20 for succinate, and m/z 144.91 > 100.97 for 2-oxoglutarate (2-OG) [67].

Chromatographic resolution was achieved using HSS T3 (2.1 × 100 mm, 1.8 μm particle size, Waters) for the TCA and the flow rate was 0.3 mL/min [68].

### Cardiotoxin injury model

For in vivo cardiotoxin experiments 35 μM working solution of cardiotoxin (CTX, Latoxan^®^, France) is prepared by diluting the stock solution in sterile phosphate-buffered saline (PBS). Mice were anesthetized with Isofluorane and both tibialis, gastrocnemius and quadriceps (just for 7 and 14 days) muscles were injected with 10, 30, and 90 µl respectively. The muscles were harvested after 3,7 or 14 days. Injections were blinded to the experimental mouse genotypes.

### Flow cytometry

BMDM from OPA1^f/f^ and OPA1^M/M^ were collected with PBS-EDTA 2 mM and Fc-receptor block (anti-mouse CD16/32, BD) was performed before the stain protocol for flow cytometric analysis with the following antibodies (Supplementary Table 3), for 20 min at 4 °C. The samples were acquired with a BD FACSCelesta (Beckman Coulter), and data were analyzed with FlowJo V10.0 Software.

### Statistical analysis

Data are reported as the mean ± SEM of at least 3 independent experiments (the specific number is indicated in the figure legends for each experiment). Statistical analysis was performed using GraphPad Prism 6 (GraphPad Software). Comparisons between two groups were performed by the nonparametric Mann–Whitney U test or Kruskal–Wallis test for multiple comparisons with Dunn’s post hoc test *P < 0.05, **P < 0.01, ***P < 0.001, ****P < 0.0001.

## Supplementary information

Supplementary Figures

Supplementary Tables

Uncropped blots

Reproducibility checklist CDD

## Data Availability

The data supporting the findings of this study are available from the corresponding authors upon written request.
