# Supplementary material for: OPA1 drives macrophage metabolism and functional commitment via p65 signaling
Source: Cell Death Differ. 2022 Oct 28;30(3):742–52. doi: 10.1038/s41418-022-01076-y (PMC9984365; doi:10.1038/s41418-022-01076-y)
Supplement: Supplementary file 1 — Supplementary Figures [file 41418_2022_1076_MOESM1_ESM.docx]

Supplementary Figures and Legends:


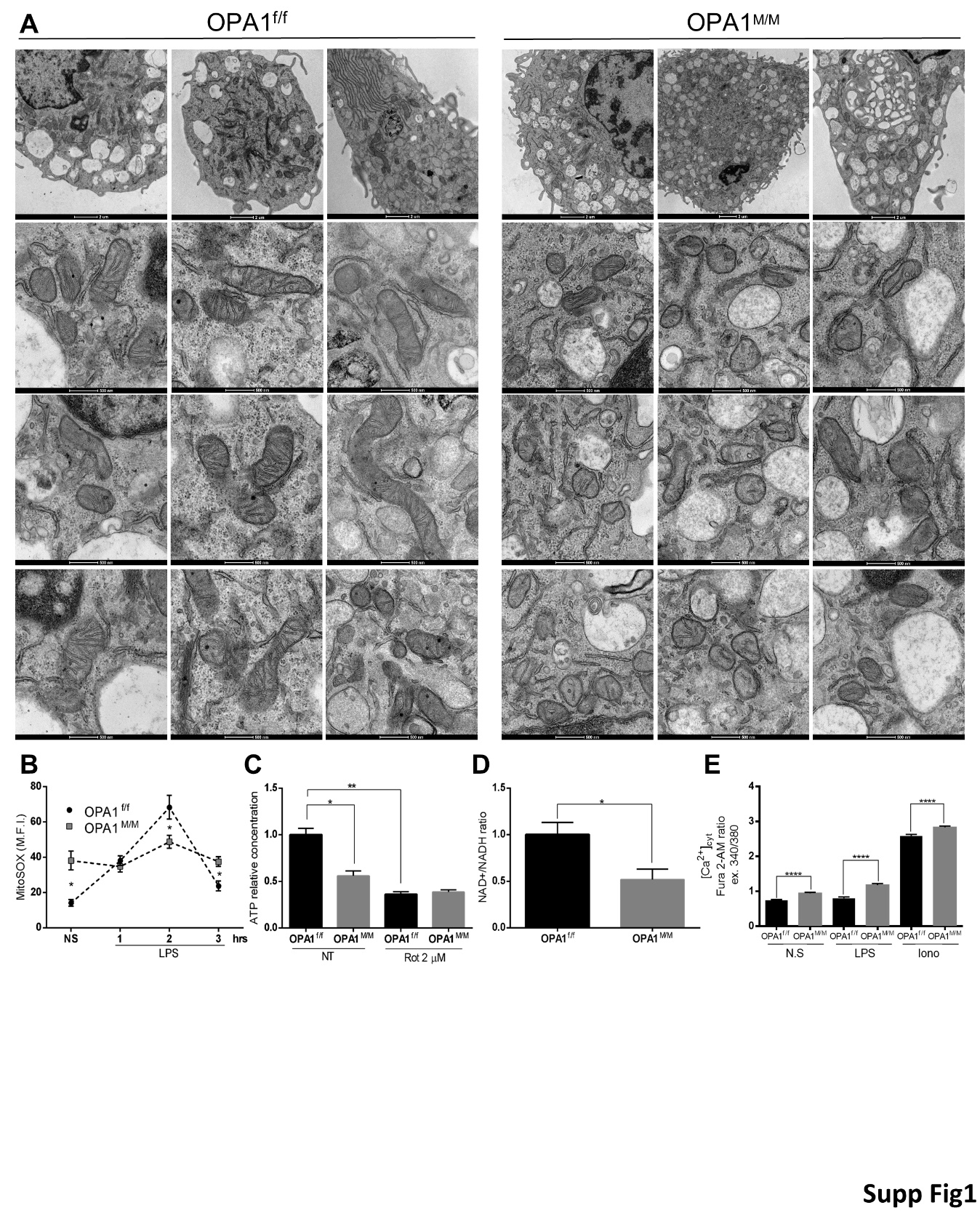


**Fig Sup 1. OPA1 deficient macrophages characterization.** BMDMs from OPA1f/f and OPA1M/M were differentiated for 7 days with 40 ng/mL of MCSF. A) Representative TEM pictures in OPA1^f/f^ and OPA1^M/M^ BMDM. Scale bar 200 nm. (n=3) B) Reactive Oxygen Species (ROS) were analysed by FACS using the MitoSOX probe before and after stimulation with LPS (500 ng/mL) for 1, 2 and 3 hours (n=3). C) Relative ATP concentration in BMDMs was measured by ATPlite Luminescence Assay. Rotenone (2 µM) addition was used as a control. D) NAD+/NADH ratio quantification and E) calcium transients by Fura-2-AM ratio (340/380) in OPA1^f/f^ and OPA1^M/M^ either unstimulated (NS), stimulated with LPS (100 ng/mL) or Ionomycin (Iono) 10 µM. Data are represented as mean ± SEM. Statistical analysis was performed by an Unpaired non-parametric t test. (*P<0.05, ** P<0.01**** P<0.0001).


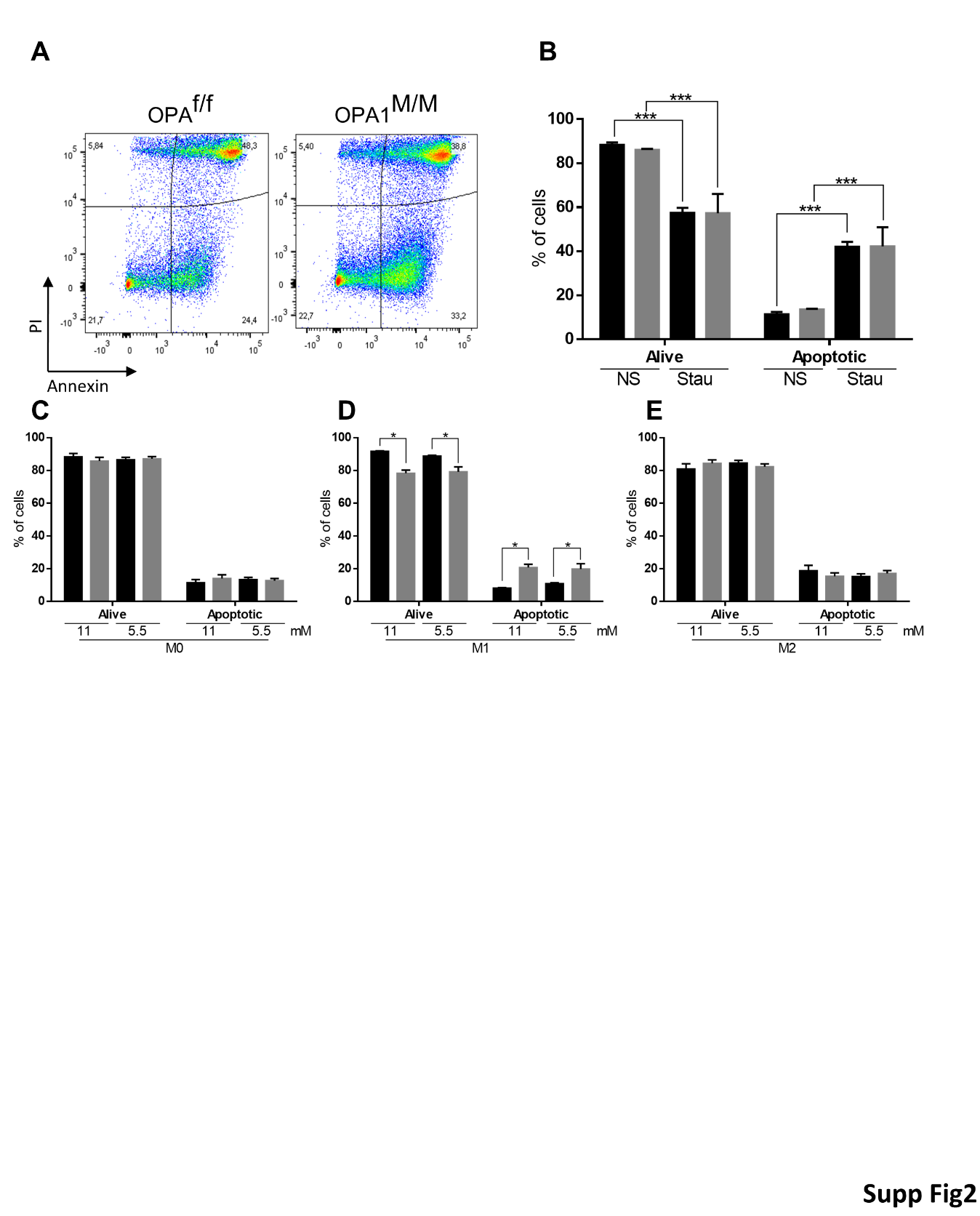


**Fig Sup 2. Cell death analysis** A) Representative Gating strategy for Annexin V assay. BMDMs from OPA1^f/f^ and OPA1^M/M^ were differentiated for 7 days with 40 ng/mL of MCSF. M0 BMDMs were either B) untreated (NS) or treated with Staurosporine (Stau, 0.5 µM) for 24 h and analysed by Annexin V assay. Graph represents % of alive (PI-/Annexin V-) or apoptotic (Annexin V +) cells (*n=4*). OPA1^f/f^ and OPA1^M/M^ BMDM were polarized to **C)** M0: MCSF (10 ng/mL); **D)** M1: LPS (500 ng/mL) and INFᵞ (25 ng/mL); **E)** M2: IL-4 (20 ng/mL) for 24 h with different concentrations of glucose (11 and 5.5 mM) and analysed with Annexin V assay. Graph represents % of cells (*n=4*). Data are represented as mean ± SEM. Statistical analysis was performed by Unpaired non-parametric *t* test (**P<0.05,* *** *P<0.001*).

**
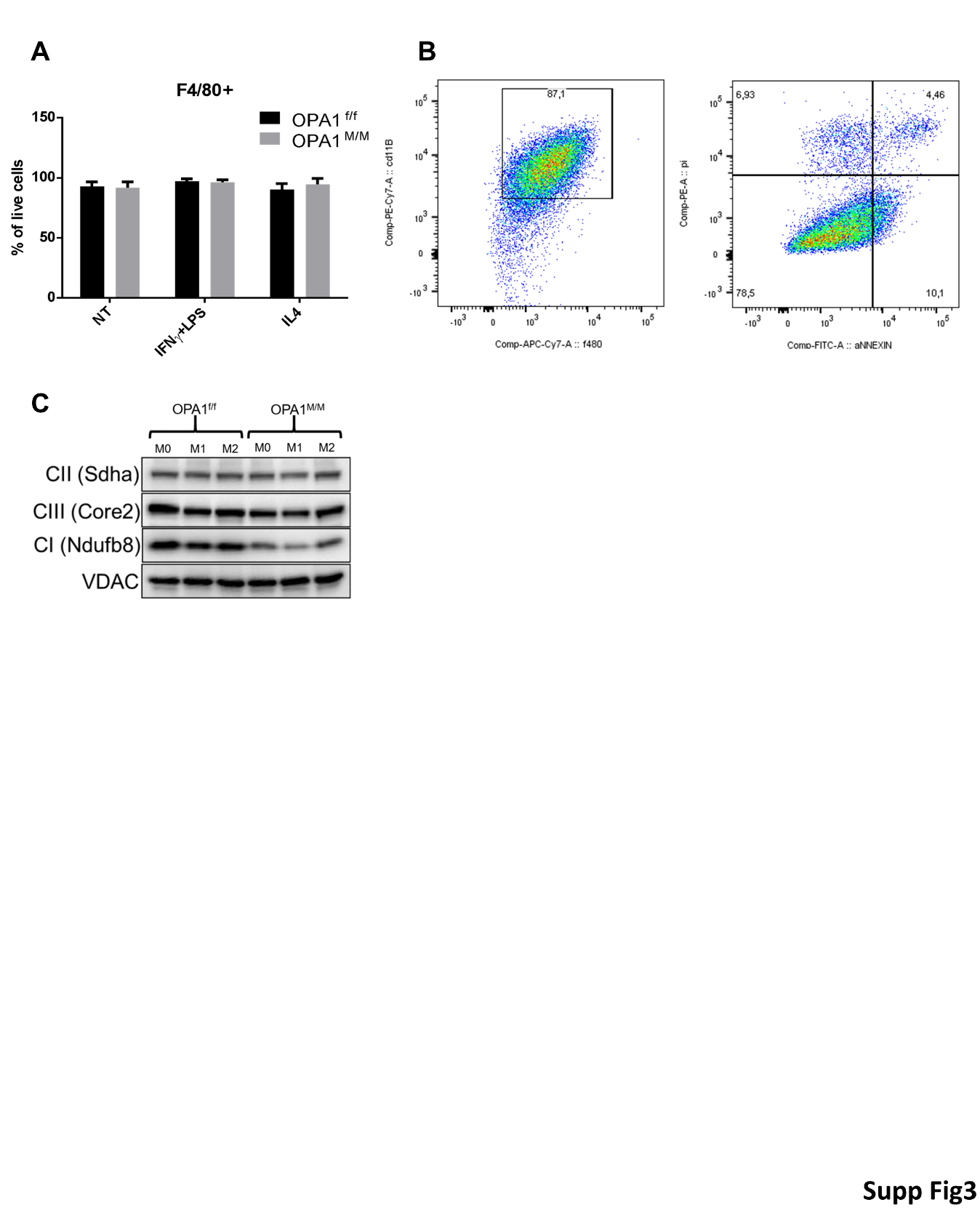
**

**Fig Sup 3. OPA1 deletion does not affect the F4/80 macrophage marker.** BMDMs from OPA1^f/f^ and OPA1^M/M^ were differentiated for 7 days with 40 ng/mL of MCSF**,** then were stimulated with LPS (500 ng/mL) for 24 hours. **A)** Graph of percentage of cells positive for macrophage markers after differentiation and **B)** Gate strategy. **C)** Representative western blot for mitochondrial proteins complex I (Ndufb8) Complex II (SDHA) and complex III (Core2). VDAC was used as a loading control. Data are represented as mean ± SEM. Statistical analysis was performed by an Unpaired non-parametric *t* test.


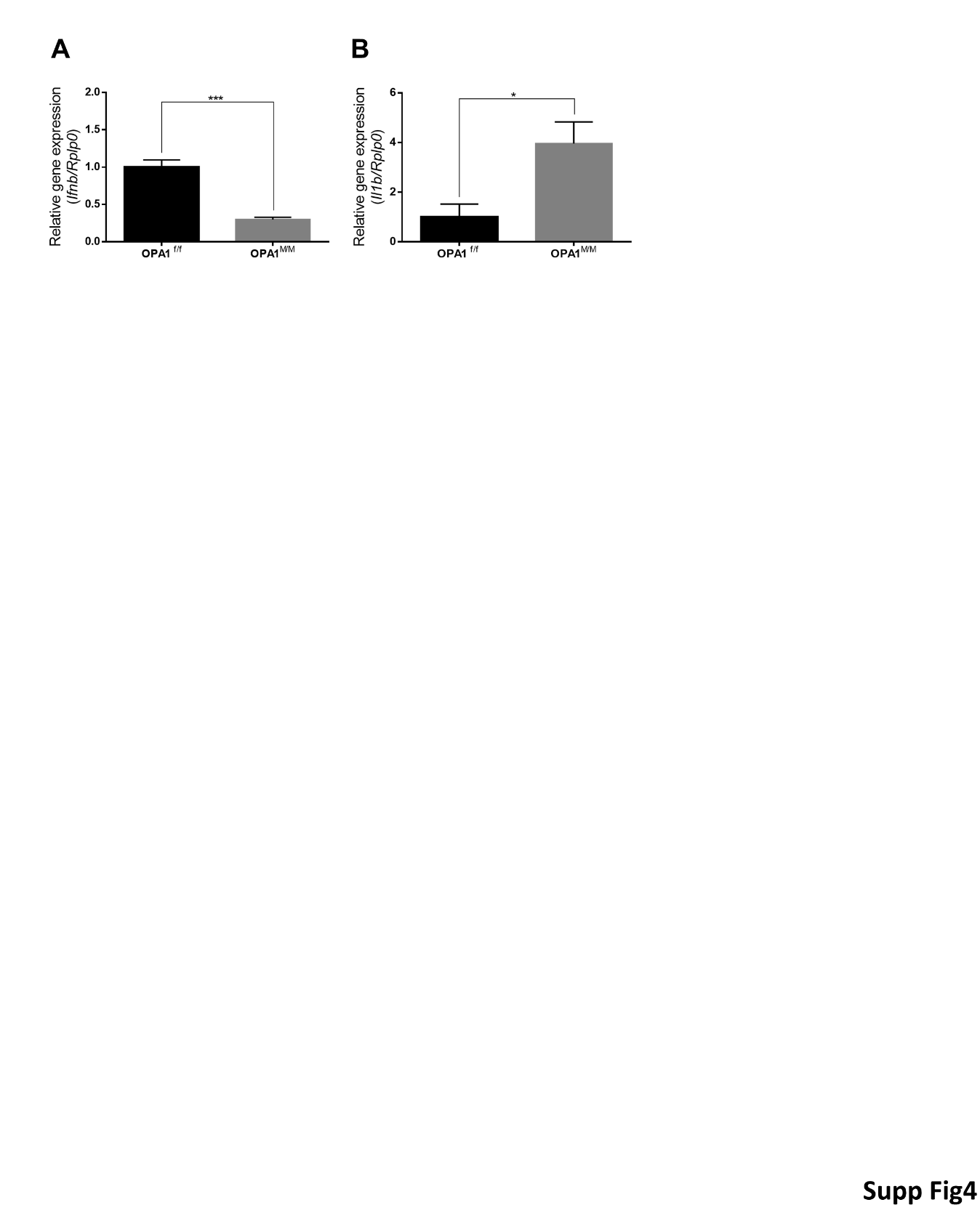


**Fig Sup 4.** **OPA1 deletion impairs M1 polarization.** OPA1^f/f^ and OPA1^M/M^ BMDMs were differentiated with MCSF (40 ng/mL) for 7 days, then BMDMs were treated with LPS (500 ng/mL) and INFᵞ (25 ng/mL) to induce the M1 polarization. Relative gene expression for M1 polarization markers **A)** Ifnb and **B)** Il1b. Rplp0 was used as housekeeping in all the experiments (*n=5*). Data are presented as mean ± SEM. Statistical analysis was performed by Unpaired non-parametric *t* test (**P<0.05,* *** *P<0.001*).

.
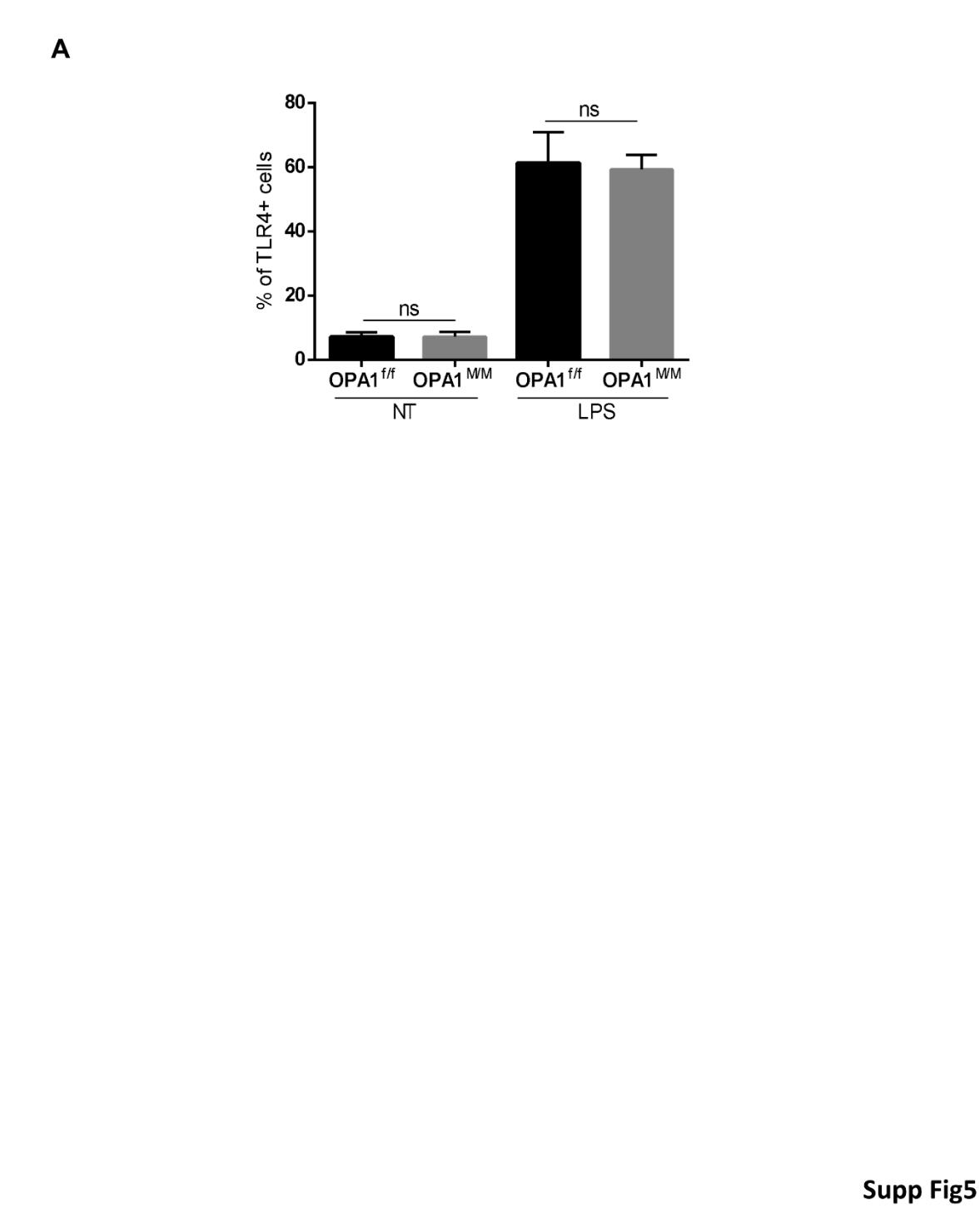


**Fig Sup 5. OPA1 deletion does not affect TLR4 expression.** BMDMs from OPA1^f/f^ and OPA1^M/M^ were differentiated for 7 days with 40 ng/mL of MCSF**,** then were stimulated with LPS (500 ng/mL) for 24 hours. **A)** Percentage of TLR4 positive cells. Data are represented as mean ± SEM.


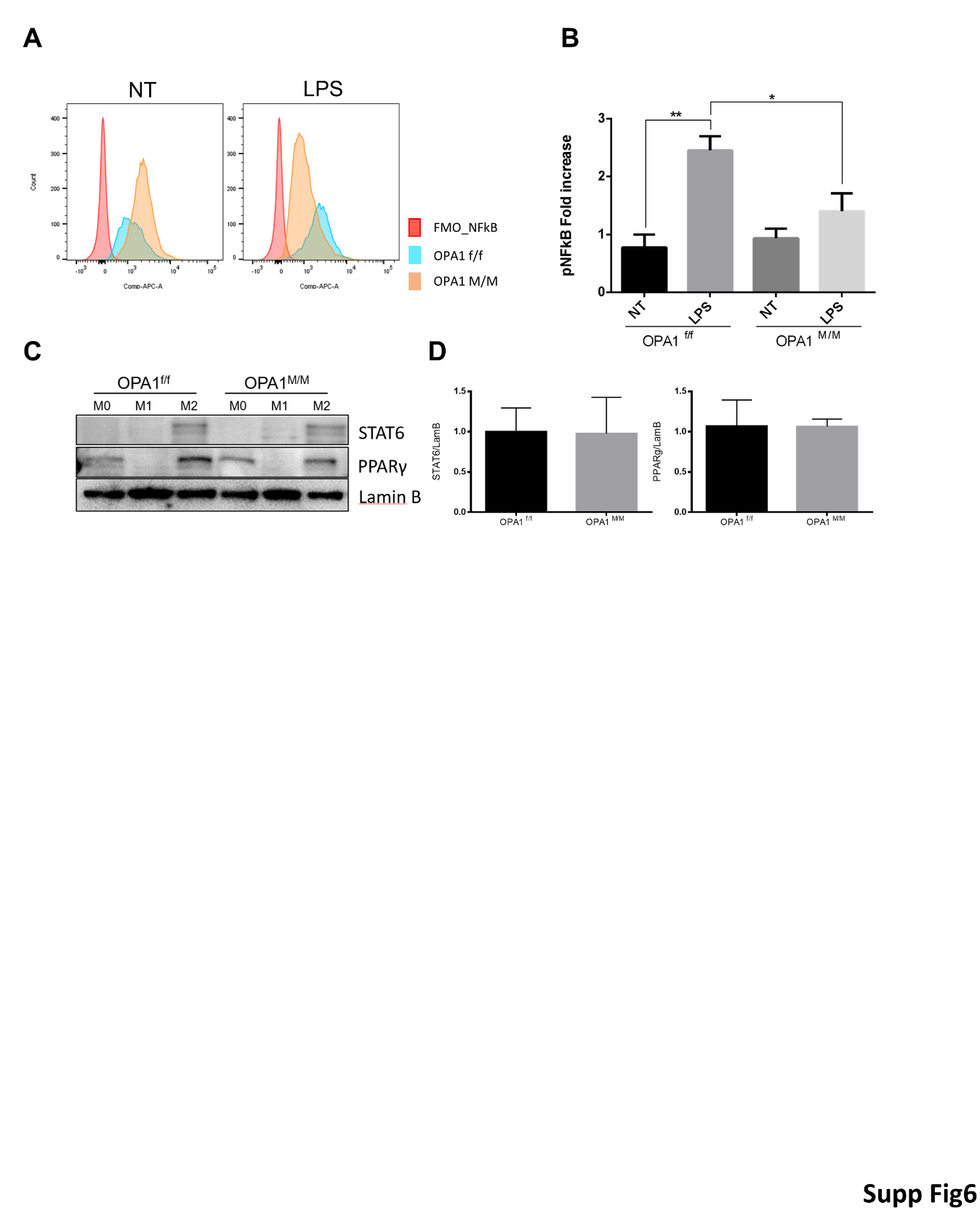


**Fig Sup 6. OPA1 deletion impairs NF-κB activation.** BMDMs from OPA1^f/f^ and OPA1^M/M^ were differentiated for 7 days with 40 ng/mL of MCSF, cells were polarized M0: MCSF (10 ng/mL); M1: LPS (500 ng/mL) and INFᵞ (25 ng/mL); M2: IL-4 (20 ng/mL) for M2 for 24 h. FACS analysis for NF-κB activation before and after LPS (500 ng/mL) stimulation **A)** density plot and **B)** fold induction of NF-κB activation. **C)** Representative western blot from nuclear protein and **D)** quantification for STAT6 and PPARϒ. LaminB (LamB) was used as a loading control. Data are represented as mean ± SEM. Statistical analysis was performed by Unpaired non-parametric *t* test. (**P<0.05,* ** *P<0.01*).


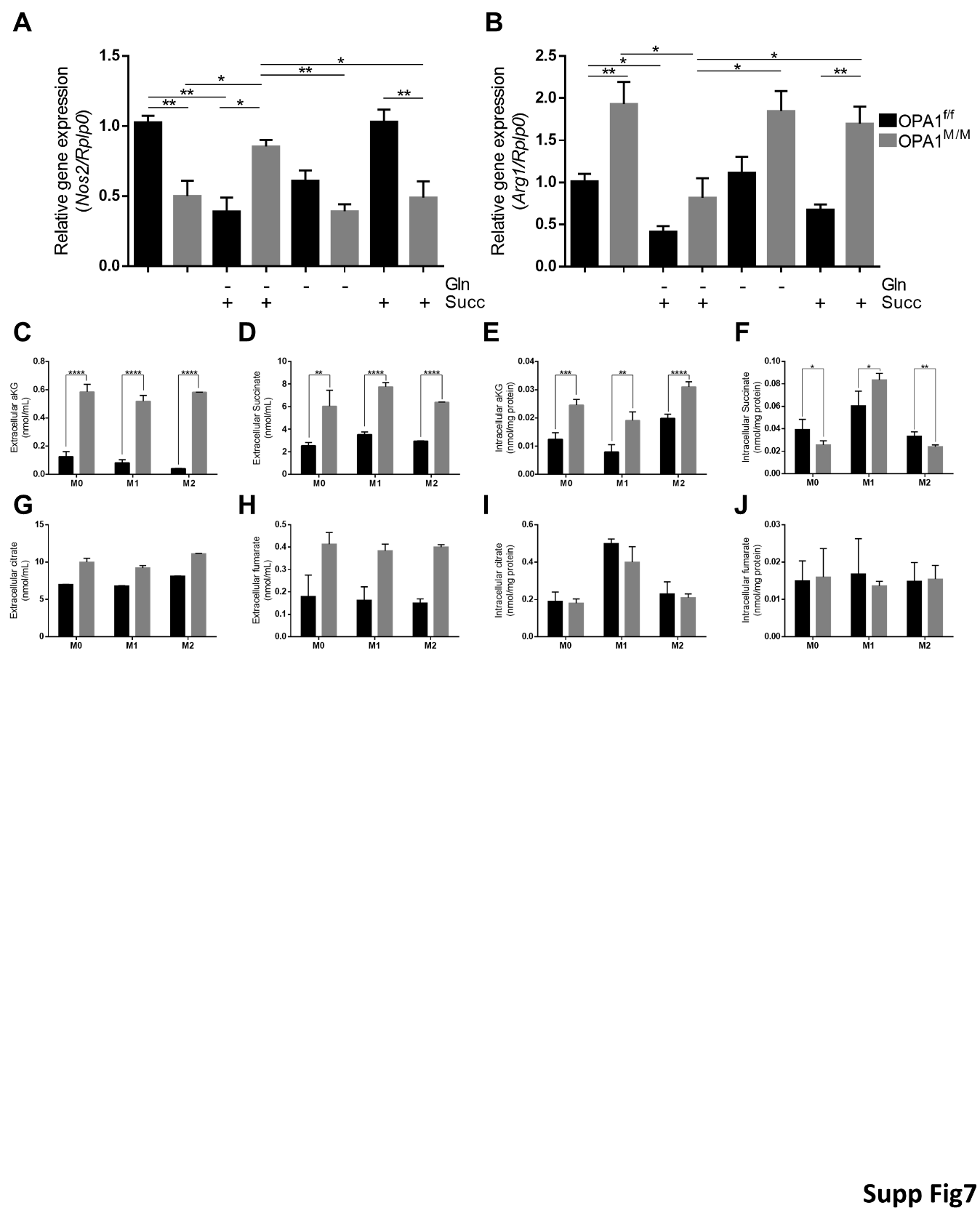


**Fig Sup 7. OPA1 deletion increase the TCA intemediates.** BMDM were differentiated to M1 or M2 in medium with Glutamine (Gln) 300 mg/L, without Gln (-) or supplemented with Dimethylsuccinate (Succ/+) 5 mM. Relative gene expression for **A)** Nos2 and **B)** Arg1 BMDMs from OPA1^f/f^ and OPA1^M/M^ were differentiated for 7 days with 40 ng/mL of MCSF, cells were polarized M0: MCSF (10 ng/mL); M1: LPS (500 ng/mL) and INFᵞ (25 ng/mL); M2: IL-4 (20 ng/mL) for M2 for 24 h. Quantification of TCA intermediates by mass spectrometry LC-MS/MS analysis from (**C-D and G-H**) supernatant and (**E-F and I-J**) cell lysate in M0, M1 and M2 macrophages. Data are presented as mean ± SEM. Statistical analysis was performed by Unpaired non-parametric *t* test (**P<0.05,* ** *P<0.01,* *** *P<0.001,***** *P<0.0001*).


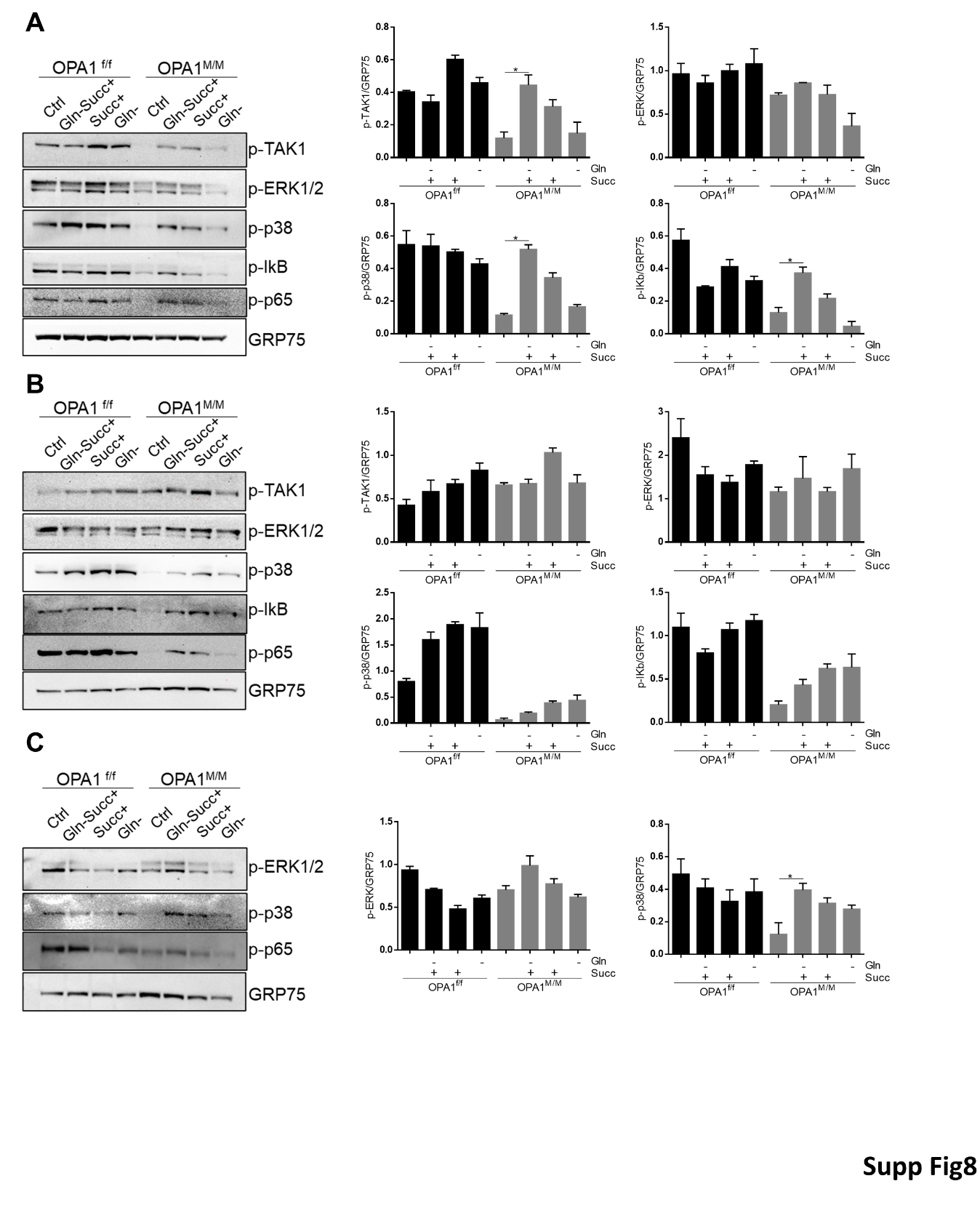


**Fig Sup 8. OPA1^M/M^ macrophages showed a TLR4-NF-Κb defective signalling pathway.** BMDMs from OPA1^f/f^ and OPA1^M/M^ were differentiated for 7 days with 40 ng/mL of MCSF. During the M1 stimulation with LPS (500 ng/mL) and INFᵞ (25 ng/mL) for 24 h, cells were cultured in medium containing either Glutamine (Gln) 300 mg/L, or without Gln (-) supplemented or not with Dimethylsuccinate (Succ/+) 5 Mm. total protein was blotted for p-TAK1, p-ERK1/2, p-p38, p-IkB and p-p65 at different time points. Representative images of western blot and quantification at A) 30 minutes, B) 2 hours and C) 4 hours after stimulation (*n=4*). Data are presented as mean ± SEM. Statistical analysis was performed by Unpaired non-parametric t test (**P<0.05*).

**
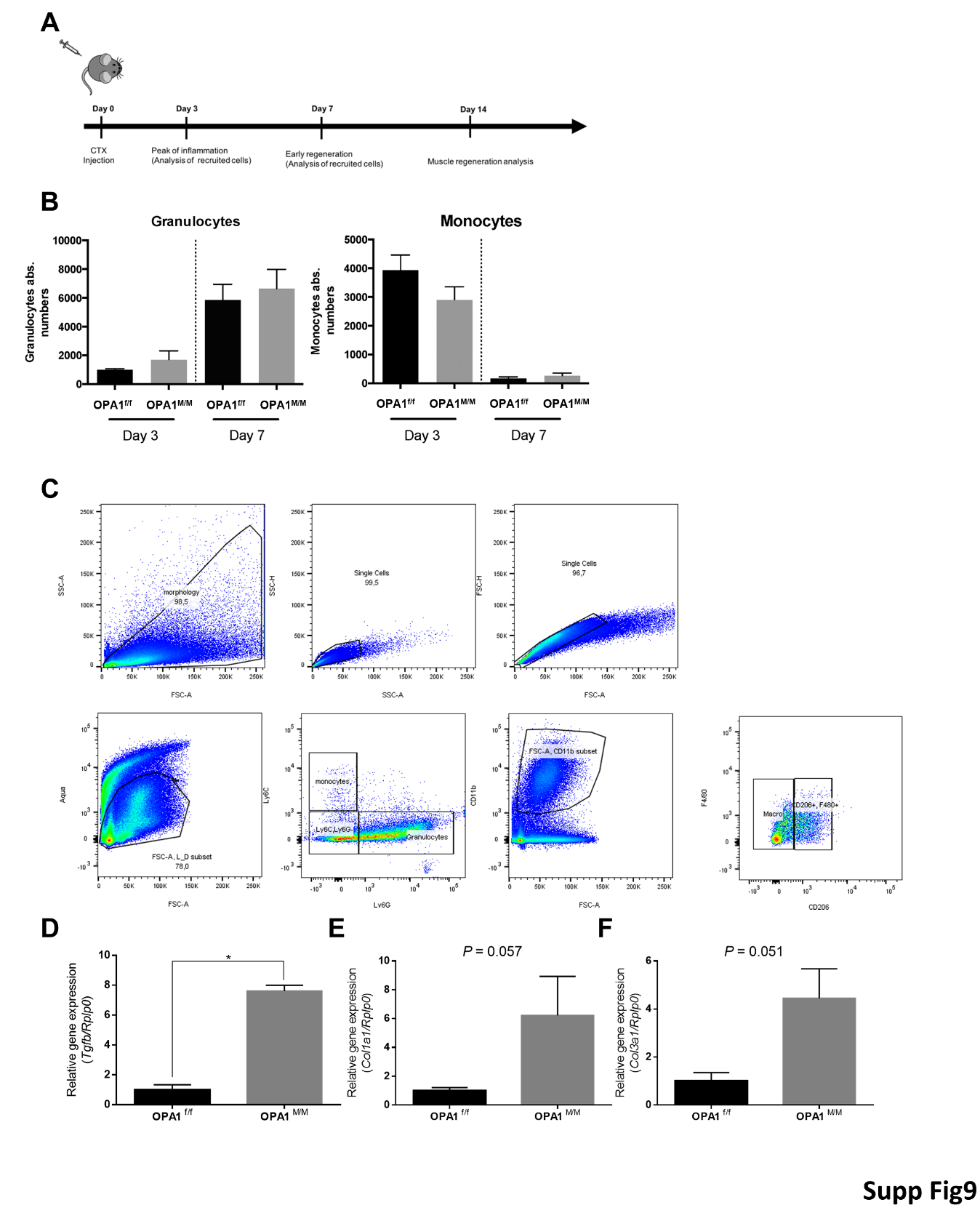
**

**Fig Sup 9. OPA1 deletion does not affect monocytes and granulocytes during muscle regeneration.** Muscle injury was induced by Cardiotoxin (35μM) injection in the gastrocnemius, tibialis and quadriceps muscles in OPA1^f/f^ and OPA1^M/M^ mice. **A)** administration and tissue collection schedule. **B)** monocyte and granulocyte quantification were performed by FACS analysis at day 3 and 7 post-injury (*n=4*). **C)** Gating strategy. Relative gene expression for **D)** Tgfb **E)** Col1a1 and **F)** Col3a1 by real-time RT-PCR. Rplp0 was used as housekeeping in all the experiments (*n=4*). Data are represented as mean ± SEM. Statistical analysis was performed by Unpaired non-parametric *t* test (**P<0.05*)
