# Supplementary material for: OPA1 drives macrophage metabolism and functional commitment via p65 signaling
Source: Cell Death Differ. 2022 Oct 28;30(3):742–52. doi: 10.1038/s41418-022-01076-y (PMC9984365; doi:10.1038/s41418-022-01076-y)
Supplement: Supplementary file 2 — Supplementary Tables [file 41418_2022_1076_MOESM2_ESM.docx]

| Supplementary Table 1. Primers used in this study | | |
| --- | --- | --- |
| Target | Forward | Reverse |
| *Opa1* | ATACTGGGATCTGCTGTTGG | AAGTCAGGCACAATCCACTT |
| *Rplp0* | GGGCATCACCACGAAAATCTC | CTGCCGTTGTCAAACACCT |
| *Nos2* | GCACATTTGGGAATGGAGACTG | GGCCAAACACAGCATACCTGA |
| *Il6* | AGGATACCACTCCCAACAGAC | GCCATTGCACAACTCTTTTCTC |
| *Arg1* | ACAAGACAGGGCTCCTTTCAG | GGCTTATGGTTACCCTCCCG |
| *Mrc1* | TTGCACTTTGAGGGAAGCGA | CCTTGCCTGATGCCAGGTTA |
| *Col1a1* | GATTGAGAACATCCGCAGCC | TACTCTCCGCTCTTCCAGTCA |
| *Col3a1* | ACGTAAGCACTGGTGGACAG | CAGGAGGGCCATAGCTGAAC |
| *Retnla* | CCTGCTGGGATGACTGCTAC | CAGTGGTCCAGTCAACGAGT |
| *Ifnb* | GCAGCTGAATGGAAAGATCA | GTGGAGAGCAGTTGAGGACA |
| *Tgfb* | CAGACAGAAGTTGGCATGGTAG | TATTGCTTCAGCTCCACAGAGA |

| Supplementary Table 2. Antibodies used for Western blot analysis | | |
| --- | --- | --- |
| Target | Brand | Cat. N |
| OPA1 | BD | 612606 |
| NDUFB8 | Abcam | ab192878 |
| SDHA | Abcam | ab14715 |
| Core2 | Abcam | ab14745 |
| NOS2 | Cell Signaling | 13120 |
| ARG1 | Santa Cruz Bio | sc-166920 |
| GAPDH | Santa Cruz Bio | sc-166574 |
| p-IKKα/β | Cell Signaling | 2697T |
| NFkB (p65) | Cell Signaling | 3033 |
| LaminB | Abcam | ab8982 |

| Supplementary Table 3. Antibodies used for FACS analysis | | |
| --- | --- | --- |
| Target | Brand | Cat. N |
| CD11b | BD | #550993 |
| F4/80 | Miltenyl | #130-102-193 |
| Ly6C | eBioscience | #48-5932-82 |
| Ly6G | BD | #5660601 |
| CD206 | BioLegend | #141712 |
| TLR4 | BioLegend | #117606 |
