# Supplementary figures and images for: OPA1 drives macrophage metabolism and functional commitment via p65 signaling
Source: Cell Death Differ. 2022 Oct 28;30(3):742–52. doi: 10.1038/s41418-022-01076-y (PMC9984365; doi:10.1038/s41418-022-01076-y)

Figure 1

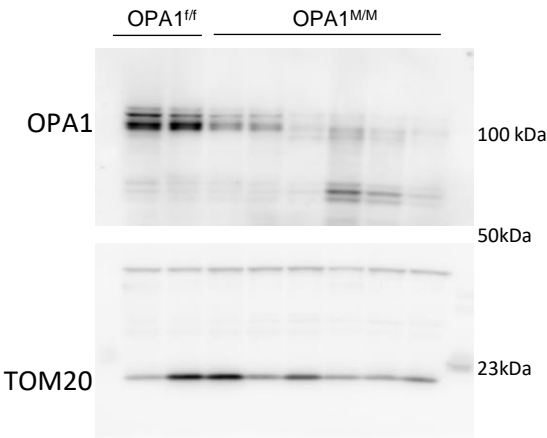

Figure 3

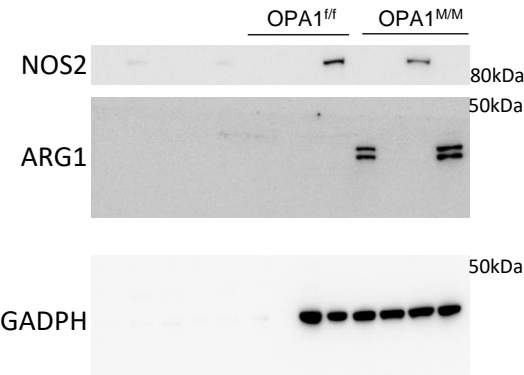

Figure 4

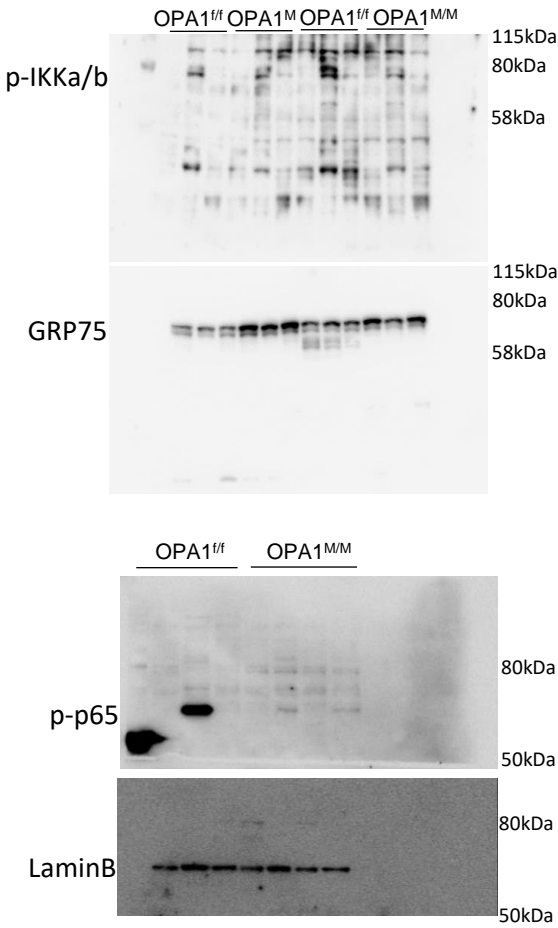

Figure 5

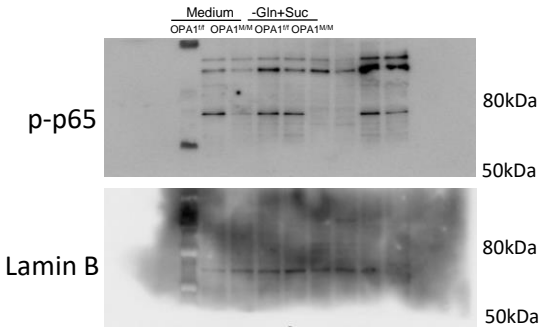

Supplement: Supplementary file 3 — Uncropped blots [file 41418_2022_1076_MOESM3_ESM.pdf]
